# An Example Worthy of Imitation

**Published:** 1865-07

**Authors:** 


### EDITORIAL AND MISCELLANEOUS.

### AN EXAMPLE WORTHY OF IMITATION.

The Chicago *Republican*, edited by the Hon. C. A. Dana, well known as one of the former editors of the New York *Tribune*, and more recently as Assistant Secretary of War, in the sixth number of its first volume, contains the following praiseworthy announcement:

"IMPROPER ADVERTISEMENTS—*An Effort to Abate an Offence Against Morality and Public Decency.*—After a careful examination of a certain class of advertisements that, we regret to say, have, for the past few numbers, in imitation of a large portion of the secular press, been published in our paper, the publisher has to-day issued an order that hereafter not one single column or line of matter shall be printed upon the pages of the *Republican* as an advertisement, or otherwise, that cannot be perused without a blush by the most refined and sensitive reader.

In adopting this course we appeal to the moral sense of the community for approval and support.

The advertisements that now crowd the "Special Notice" column of our papers are a stench and an abomination in the nostrils of all decent men, and ought to debar the paper that contains them from admission within the pure and sacred precincts of the family circle.

The foul pollution that springs from social crimes should flow off through the deepest and darkest sewers, and not be permitted to run through every public hall and private room and upon the surface of every public avenue. By thus excluding this noxious class of advertisements from the columns of the *Republican* we may diminish somewhat its revenues, but will at least have the satisfaction of printing a paper that can be read without offence by every moral and religious member of society."

Our attention being in this way called to the subject, we have looked over the secular press of this city more attentively than before, the better to judge of the character of what the *Republican* has determined to exclude; and we will say, we were seldom more surprised than when we read the obscene, disgusting and immoral advertisements that papers claiming to be respectable place conspicuously in their columns. Not to speak of the taste and propriety—what shall we say of the morality—of permitting any of either sex, but especially the young, and the pure-minded females of Chicago, to read the loathsome details found in the advertisements under such heads as "The Key to Love," "Highly important to Married People," "Philosophy ot Marriage," "The Young Man's Friend"—the filthy advertisements ot those who treat private diseases, and of medicines to re-establish suppressed menstruation, accompanied with an italicized caution to ladies not to use them when there is any suspicion of pregnancy, as they will be sure to produce abortion. Persons thus advertising are known by the conductors of these periodicals to be charlatans and imposters—such medicines are known to be advertised as agents for producing abortion, and yet the spirit of avarice, the greed to add lot to lot and house to house, gives them place. We have known death to follow as the result of using these advertised agents of abortion, and can we hold those who give currency to the advertisement, influenced only by venal motives, as guiltless of the crime? They have no shelter under the published caution against such use of the medicines. As well might one who should furnish something known to be designed for murder or suicide, hope to escape complicity in guilt by labeling the substance poison.

If a sense of propriety, morality and right will not restrain this class of papers, the community should appeal to their pecuniary sensibilities, and demand that such revolting matters should be excluded from their pages, or expel them from the abodes of virtue, to the brothels for which they would seem designed, and where they might be read, if not with approval, at least without a blush.

